# Association of breastfeeding and three-dimensional dental arch relationships in primary dentition

**DOI:** 10.1186/s12903-015-0010-1

**Published:** 2015-03-10

**Authors:** Fung Hou Kumoi Mineaki Howard Sum, Linkun Zhang, Hiu Tung Bonnie Ling, Cindy Po Wan Yeung, Kar Yan Li, Hai Ming Wong, Yanqi Yang

**Affiliations:** 1 Paediatric Dentistry and Orthodontics, Faculty of Dentistry, The University of Hong Kong, 34 Hospital Road, Sai Ying Pun, Hong Kong SAR China; 2 Department of Orthodontics, Tianjin Stomatological Hospital of Nankai University, 75 Dagu Road, Tianjin, China; 3 Translational Research Laboratory, Faculty of Dentistry, The University of Hong Kong, 34 Hospital Road, Sai Ying Pun, Hong Kong SAR China

**Keywords:** Breastfeeding, Occlusion, Primary dentition

## Abstract

**Background:**

The benefits of breastfeeding on oral health are still inconclusive, especially the association on occlusion. This study aimed to investigate the association of breastfeeding and the development of primary dentition.

**Methods:**

A cross-sectional study was conducted with 851 Asian children aged 2–5 years old in Hong Kong. Questionnaires were completed by the parents to collect information on breastfeeding and the non-nutritive sucking habits. The children’s dental arch relationships were examined in the sagittal, vertical, and transverse dimensions by an experienced examiner.

**Results:**

Children who experienced pure breastfeeding for more than 6 months had a lower chance of developing a class II incisal relationship (*P* < 0.05) or an increased overjet (*P* < 0.05), and had wider intercanine (*P* < 0.05) and intermolar widths (*P* < 0.05). Vertically, no association on the extent of overbite or openbite was found (*P* >0.05).

**Conclusions:**

Pure breastfeeding for more than 6 months is positively associated with primary dental arch development in the anterior sagittal dental segment and on the horizontal arch width in primary dentition. Therefore, pure breastfeeding for more than 6 months is recommended, as it is associated with lower chance of the development of abnormal dental relationships. The results will be valuable for education and promotion of maternal breastfeeding.

**Electronic supplementary material:**

The online version of this article (doi:10.1186/s12903-015-0010-1) contains supplementary material, which is available to authorized users.

## Background

Breastfeeding is the ideal mode of feeding for newborns and infants. It provides infants with all of the nutrients they need. In the short term, the antibodies in breast milk protect infants from common childhood diseases such as diarrhea and pneumonia [1,2]. In the long term, adolescent and adults that were breastfed as babies have a lower chance of being obese or overweight and of developing type 2 diabetes [1,2]. Breastfed children also perform better in intelligence tests [1,2].

There is no doubt that breastfeeding has benefits for general health; nevertheless, the relationship of breastfeeding on oral health is still inconclusive. Although the growth and development of the facial bones is strongly associated with genetic factors [3], it is also believed that environmental factors such as breastfeeding and oral parafunctional habits also affect facial growth [4].

There are controversies regarding the interaction of breastfeeding with deleterious oral habits. Most studies, systematic review and meta analysis show that pacifier use is associated with shorter duration of breastfeeding although the causal relationship cannot be identified yet [5-7]. However, in one systematic review, they conclude that the highest level of evidence does not support a relationship between pacifier use and duration of breastfeeding [8].

There is evidence that certain parafunctional oral habits have deleterious effects on dental arch development. For instance, prolonged non-nutritive sucking habits such as pacifier use and digit sucking are associated with an anterior open bite, reduced overbite, increased overjet, and posterior crossbite [9-16]. Thumb sucking is also strongly associated with a narrow maxillary arch [15,17]. However, the literature regarding the effects of breastfeeding on occlusion is inconclusive [1]. The American Academy of Pediatric Dentistry and the American Academy of Pediatrics have called for more research into the effects of breastfeeding on dentofacial and oral development to set appropriate policy on breastfeeding [18].

Even though certain studies find that a significantly larger percentage of straight terminal planes were recorded in the children who are entirely breastfed when compare to children that’s exclusively bottle-fed, and that breastfeeding children would have a lower chance of developing anterior open bite and posterior crossbite, one review article published in the Journal of the American Dental Association in February 2013 discussed the breastfeeding and oral health, but did not conclude whether or not breastfed children develop more favorable occlusion in primary dentition [1,5,19,20].

As some of the occlusal status of primary dentition is believed to be important to the occlusal development of permanent dentition, the objective of this study was to investigate the association of maternal breastfeeding on the intra-arch and inter-arch dental relationships in primary dentition [21].

## Methods

### Samples

A cross-sectional study was carried out with ethical approval from Institutional Review Board of the University of Hong Kong/Hospital Authority Hong Kong West Cluster (HKU/HA HKW IRB: UW12-334). Using cluster sampling method, invitations were sent to 17 kindergartens (all the kindergartens under the same education association) in the three main territories of Hong Kong: Hong Kong Island, Kowloon and the New Territories. Ten kindergartens (distributed across the three territories) agreed to participate in the study. Written consents were obtained from the parents/guardians for participation in the study and examination of their children. All the children (n = 1014) in the kindergartens were invited to participate in the study. Some subjects were lost (n = 24) in the study either due to the parents did not complete or return the questionnaires or the parents did not sign the consent form. Other subjects were excluded as they were uncooperative during clinical examinations or refused clinical examinations. Non-Asians were not included in the study. Subjects with orofacial cleft, distinct facial deformities, congenital defect or systemic disease were also excluded in this study. A total of 851 Asian children (469 boys, 378 girls and 4 with unreported gender) aged 2–5 (3.42 ± 1.10) years old were finally recruited with written parental consent. Among the 10 kindergartens, the social economic status including their income and family backgrounds was assessed by the questionnaire evaluation. The results between the kindergartens are similar with most of them are from below mediocre family. In addition, there was no difference between the included and excluded data in terms of monthly family income (Chi Square P = 0.818).

In a previous Italy study of 3 to 6-year-old children, the estimated mean intermolar width ranged from 27.8 mm to 29.6 mm with a standard deviation of 1.3 mm to 2.2 mm, and the estimated mean intercanine width ranged from 21.6 mm to 22.5 mm with a standard deviation of 1.2 to 1.8 [22]. Thus, by assuming the standard deviation to be 2.2 and an even difference distribution across the groups, it was estimated that a sample of 285 individuals would have an 80% chance of detecting a 1 mm mean difference in width among the different breastfeeding habit groups with an allocation ratio of 1 at the two-sided significance of 0.05. In another study in Taiwan looking at the intermolar width of 4 to 5-year-old children, the mean intermolar width ranged from 33.2 mm to 34.7 mm with a standard deviation of 1.6 to 1.7 mm [23]. Thus, by assuming the standard deviation to be 1.7 and an even distribution across the groups, it was estimated that a sample of 171 individuals would have an 80% chance of detection a 1 mm mean difference in width among the different breastfeeding habit groups with an allocation ratio of 1 and also the two-sided significance level of 0.05.

For the logistic regression, by assuming the probability of the event (dental arch development) to be 0.1 and the allocation ratio to be 1, it was estimated that a sample of 487 individuals would have an 80% chance of detecting an odds ratio of 0.6 with the two-sided significance level setting at 0.05.

Given these two sample size determinations and assuming 20% possible non-responses and losses, the final study population had to be at least 609. As the study recruited 851 subjects, the sample size was sufficient.

### Data collection

Questionnaires were completed by the parents to collect information on the children’s duration of full-time breastfeeding, history of non-nutritive sucking habits (thumb sucking and pacifier use), as well as the family social economic status including their income and family background. An additional text file shows this in more detail (see Additional file 1).

Oral examination was performed in a standardized manner throughout the study by the same examiner, who had over 5 years’ of clinical orthodontic treatment experience, and the examination standard was calibrated with another experienced orthodontist. To check the intra-examiner reliability, in accordance with the recommendation of World Health Organization (WHO) that each examiner should perform duplicate examinations on 5-10% of the sample [24], 6.23% of the sample was re-examined at the same visit, and Cohen’s kappa coefficient and interclass correlation coefficient (ICC) were analyzed [25,26]. Cohen’s kappa coefficients ranged from 0.70-1.00, indicating that the data were in substantial to perfect agreement [25]. The ICC values ranged from 0.89-0.98, indicating that the continuous data had excellent reproducibility [26].

Clinical examinations were performed in the school setting, with the child lying down under an adequate light source. The children were manipulated into centric occlusal relationship. Orthodontic ruler and hand mirror with light were used to perform the measurements. All the measurements were in millimeters.

The children’s dental arch relationship was examined in the following three dimensions with the following criteria:

I. Sagittal dimension (antero-posterior dimension)
  Incisal relationship
    o Three forms of occlusion were considered: Class I, the lower incisor edges occlude with or lie immediately below cingulum plateau of the upper central incisor. Class II, the lower incisor edges lie posterior to the cingulum plateau of the upper incisors. Class III, the lower incisor edges lie anterior to the cingulum plateau of the upper incisors. The overjet is reduced or reversed [27].
  Primary canine relationship
    o Three forms of occlusion were considered: Class I, the cusp tip of the maxillary primary canine tooth is in the same vertical plane as the distal surface of the mandibular primary canine. Class II, the cusp tip of the maxillary primary canine tooth is mesial to the distal surface of the mandibular primary canine. Class III, the cusp tip of the maxillary primary canine tooth is distal to the distal surface of the mandibular primary canine [28].
  Primary molar relationship
    o Three forms of occlusion were considered: Flush terminal plane, the distal surfaces of maxillary and mandibular primary second molars lie in the same vertical plane. Distal Step, the distal surface of the mandibular primary second molar is distal to that of the maxillary primary second molar. Mesial step, the distal surface of the mandibular primary second molar is mesial to that of the maxillary primary second molar [28].
  Overjet
    o The distance along a horizontal plane between the incisal edge of the labial surface of the mandibular central incisor and the incisal edge of the labial surface of the most labially positioned maxillary central incisor [29].
    o Overjet greater than 3.5mm was regarded as increased [30].
  Anterior crossbite
    o When one or more of the maxillary incisors occluded lingual to the mandibular incisors [29].
II. Vertical dimension
  Anterior Openbite
    o The vertical distance between the incisal edge of the maxillary and mandibular central incisors [29].
  Overbite
    o Vertical overlap between the incisal edge of the maxillary central incisor and the incisal edge of the mandibular central incisor [9].
III. Transverse dimension (side-to side dimension, i.e., arch width)
  Intercanine width
    o Distance from cusp tip to cusp tip of the maxillary primary canines [9].
  Intermolar width
    o Distance between mesiobuccal cusp tips of the maxillary second primary molars [9].
  Posterior crossbite
    o When one or more of the maxillary primary canines or molars occluded lingual to the buccal cusps of the opposing mandibular teeth [29].

Duration of exclusive breastfeeding was grouped into more than 6 months, 0 to 6 months and never because WHO advocate exclusive breastfeeding up to 6 months of age [2].

### Statistical analysis

Statistical analysis was carried out with the Statistical Package for the Social Sciences (SPSS) (IBM) version 20.0. Different types of dental arch relationships were treated as the main dependent variables, and factors including the duration of breastfeeding, age, gender, and non-nutritive sucking habits including pacifier use and thumb sucking were used as the independent variables.

For binary outcome variables, multivariable logistic regression models were used to investigate their association with different breastfeeding duration groups adjusted for age, gender, pacifier use and thumb sucking habits.

For the categorical variables, including incisal relationship, canine relationship and molar relationship in primary dentition, multinomial logistic regression models were used to investigate their associations with different breastfeeding duration groups adjusted for age, gender, pacifier use and thumb sucking habits.

For intercanine and intermolar width, multi-way ANOVA with Bonferroni adjustment of pairwise comparisons was used to compare the mean intercanine width and mean intermolar width among different breastfeeding duration groups adjusted for the potential categorical confounders (age, gender, pacifier use and thumb sucking habits).

The level of statistical significance was set at 0.05 and the tests were two-sided tests.

## Results

### Characteristics of the study group

A total of 851 children were recruited into the study. The age range of the subjects was 2–5 years old. The study recruited 20.6-27.3% of the subjects from each age group. The boys (n = 469, 55.1%) to girls (n = 378, 44.4%) ratio was 1.24.

About a quarter of the children were purely breastfed for more than 6 months (n = 212, 24.9%), and less than one third (n = 262, 30.8%) had never been breastfed. The majority of the children had experienced less than 6 months of pure breastfeeding (n = 360, 42.3%).

Most of the children had never sucked their thumb (n = 397, 46.7%) or used a pacifier (n = 449, 52.8%), but 13.3% (n = 113) of the children had sucked their thumb and 25.0% (n = 213) had used a pacifier more than a few times a week and 36.2% (n = 308) and 18.8% (n = 160) had sucked their thumb or used a pacifier less than a few times a month.

A descriptive table (Table 1) including the measures for each breastfeeding group shows that children who breastfed more tended to be associated with lower chance of developing overjet >3.5 mm. There was a tendency that longer duration of breastfeeding was associated with higher class I incisal pattern. Lower percentage of the subjects were found to have class II incisal pattern when comparing the group that were pure breastfed for more than 6 months with the group that had never been breastfed. The class III incisal pattern remained more or less the same between three groups. There were minor increment in intercanine and intermolar width of the pure breastfeeding group for more than 6 months when compared to those who had never been breastfed.

**Table 1:** **Descriptive data of the measures for each breastfeeding group**

Variables		Pure breastfed for > 6 months	Pure breastfed for ≤ 6 months	Never breastfed
Overjet*	**≤3.5 mm**	169 (88.0%)	276 (85.2%)	187 (79.2%)
	**>3.5 mm**	23 (12.0%)	48 (14.8%)	49 (20.8%)
Incisal relationship*	**Class I incisor**	126 (65.6%)	199 (60.9%)	131 (55.3%)
	**Class II incisor**	36 (18.8%)	83 (25.4%)	78 (32.9%)
	**Class III incisor**	30 (15.6%)	45 (13.8%)	28 (11.8%)
**Canine Relationship**	**Class I canine**	57 (35.8%)	76 (29.7%)	60 (30.3%)
	**Class II canine**	89 (56.0%)	157 (61.3%)	123 (62.1%)
	**Class III canine**	13 (8.2%)	23 (9.0%)	15 (7.6%)
**Molar Relationship**	**Flush**	58 (43.6%)	97 (44.5%)	75 (46.3%)
	**Mesial step**	53 (39.8%)	69 (31.7%)	47 (29.0%)
	**Distal step**	22 (16.5%)	52 (23.9%)	40 (24.7%)
**Anterior Crossbite**	**Yes**	25 (12.8%)	51 (15.5%)	26 (10.9%)
	**No**	170 (87.2%)	279 (84.5%)	213 (89.1%)
**Anterior overbite**	**≥1/2**	131 (67.5%)	232 (70.9%)	170 (71.4%)
	**<1/2**	63 (32.5%)	95 (29.1%)	68 (28.6%)
**Anterior openbite**	**Yes**	6 (3.1%)	9 (2.7%)	7 (2.9%)
	**No**	189 (96.9%)	320 (97.3%)	231 (97.1%)
**Posterior Crossbite**	**Yes**	5 (2.6%)	5 (1.5%)	2 (0.8%)
	**No**	190 (97.4%)	325 (98.5%)	237 (99.2%)
Intercanine width*		31.50 ± 2.09 mm	30.94 ± 1.91 mm	30.93 ± 2.05 mm
Intermolar width*		45.25 ± 2.49 mm	44.53 ± 2.41 mm	44.50 ± 2.71 mm

*Variables showing significant difference (*p* < 0.05) among the breastfeeding groups.

### Sagittal dimension

In terms of the antero-posterior relationship, Table 2 presented the results of a crude and multivariable multinomial logistic regression analysis for incisal relationship in primary dentition by breastfeeding duration groups. Significant association was found between duration of breastfeeding and incisal relationship (multinomial logistic regression: *P* = 0.013). The children who experienced pure breastfeeding had a significant lower chance of developing a Class II incisal relationship (adjusted Odds Ratio (OR) for “>6 months” = 0.650 [95% Confidence Interval (CI) 0.438-0.966] and adjusted OR for “0-6 months” = 0.452 [95% CI 0.277-0.739]) compared with Class I incisal relationship; the duration of pure breastfeeding had no significant difference in developing Class III incisal relationship (Table 2) compared with Class I incisal relationship. Table 3 presented the results of a crude and multivariable logistic regression analysis for overjet relationship in primary dentition by breastfeeding duration groups. The children who had experienced pure breastfeeding for more than 6 months also had a significantly lower chance of developing an increased overjet (logistic regression: *P* = 0.021; adjusted OR = 0.511 [95% CI 0.290-0.902]) (Table 3) than those who had never been breastfed in the adjusted model. The crude model of the logistic regression also had similar results.

**Table 2:** **Association between the duration of breastfeeding and incisal relationship in primary dentition**

	Crude model					Adjusted model^a^				
Variables	*P*-value	Class II incisor vs Class I incisor		Class III incisor vs Class I incisor		*P*-value	Class II incisor vs Class I incisor		Class III incisor vs Class I incisor	
		OR	95% CI	OR	95% CI		OR	95% CI	OR	95% CI
**Duration of breastfeeding**	0.022*					0.013*				
>6 months		0.700	0.479-1.024	1.058	0.629-1.781		**0.650**	**0.438-0.966**	1.053	0.604-1.836
0-6 months		**0.480**	**0.302-0.764**	1.114	0.630-1.970		**0.452**	**0.277-0.739**	1.200	0.657-2.192
Never		1	-	1	-		1	-	1	-

OR, odds ratio; CI, confidence interval.

^a^Adjusted for background information (age and gender) and related non-nutritive sucking habits (thumb sucking and pacifier use).

*P < 0.05.

Boldface data are variables with P<0.05 or OR (95%CI) <1.

**Table 3:** **Association between the duration of breastfeeding and overjet in primary dentition**

	n	%	Overjet >3.5 mm vs non overjet >3.5 mm					
			Crude model			Adjusted model^a^		
	n	%	OR	95% CI	*P*-value	OR	95% CI	*P*-value
**Duration of breastfeeding**					0.038*			0.038*
>6 months	182	25.3	**0.519**	**0.303-0.889**	0.017*	**0.511**	**0.290-0.902**	0.021*
0-6months	311	43.3	0.664	0.428-1.030	0.067	0.634	0.400-1.005	0.052
Never	226	31.4				1	-	-

OR, odds ratio; CI, confidence interval.

^a^Adjusted for background information (age and gender) and related non-nutritive sucking habits (thumb sucking and pacifier use).

*P < 0.05.

Boldface data are variables with P<0.05 or OR (95%CI) <1.

The duration of breastfeeding had no association on the primary canine relationship (multinomial logistic regression: *P* = 0.801), primary molar relationship (multinomial logistic regression: P = 0.099), or anterior crossbite (logistic regression: *P* = 0.369).

### Vertical dimension

The duration of breastfeeding was found to have no association on the extent of overbite in primary dentition (logistic regression: *P* = 0.769). In addition, there was no significant difference between subjects with and without openbites and the duration of pure breastfeeding (logistic regression: *P* = 0.276). Hence, the duration of breastfeeding was found to have no association on the vertical dimension of primary dentition.

### Transverse dimension

In terms of the anterior width of primary dentition, the duration of pure breastfeeding was significantly associated with the intercanine width (*P* = 0.003), adjusted for age, gender, and different non-nutritive sucking habits. The children who were purely breastfed for more than 6 months had a significantly higher chance of developing a greater mean intercanine width than those who were purely breastfed for less than or equal to 6 months (pairwise comparison in ANOVA: *P* = 0.010; Mean Difference = 0.541 [95% CI 0.099-0.983]) (Table 4) and those who had never been breastfed (pairwise comparison in ANOVA: *P* = 0.006; Mean Difference = 0.608 [95% CI 0.134-1.081]) (Table 4).

**Table 4:** **Pairwise comparison of the association between the intercanine width and the duration of pure breastfeeding**

Variables	Intercanine width (mm)		*P*-value^b^	95% Confidence interval for difference	
	Mean difference	Standard error			
				Lower bound	Upper bound
**Duration of breastfeeding**			0.003*		
>6 months - Never	0.608**	0.197	0.006*	**0.134**	**1.081**
>6 months - ≤6 months	0.541**	0.184	0.010*	**0.099**	**0. 983**
≤6 months - Never	0.067	0.173	1.000	−0.349	0.483

Based on estimated marginal means.

Adjusted for background information (age and gender) and related non-nutritive sucking habits (thumb sucking and pacifier use).

**The mean difference is significant at the 0.05 level.

^b^Adjustment for multiple comparisons: Bonferroni.

*P < 0.05.

Dependent variable: Intercanine width (mm).

Boldface data are the mean differences (95% CI) that are significant at the 0.05 level.

In terms of the posterior width of primary dentition, it was found that the duration of pure breastfeeding was significantly associated with the intermolar width (*P* = 0.002), adjusted for age, gender and different non-nutritive sucking habits. The children who were purely breastfed for more than 6 months had a significantly higher chance of developing a greater mean intermolar width than those who were purely breastfed for less than or equal to 6 months (pairwise comparison in ANOVA: *P* = 0.005; Mean Difference = 0.813 [95% CI 0.193-1.433]) (Table 5) and those who had never been breastfed (pairwise comparison in ANOVA: *P* = 0.006; Mean Difference = 0.857 [95% CI 0.198-1.515]) (Table 5).

**Table 5:** **Pairwise comparison of the association between the intermolar width and the duration of pure breastfeeding**

Variables	Intermolar width (mm)		*P*-value^b^	95% Confidence interval for difference	
	Mean difference	Standard error			
				Lower bound	Upper bound
**Duration of breastfeeding**			0.002*		
>6 months - Never	0.857**	0.274	0.006*	**0.198**	**1.515**
>6 months - ≤6 months	0.813**	0.258	0.005*	**0.193**	**1.433**
≤6 months - Never	0.044	0.249	1.000	−0.554	0.642

Based on estimated marginal means.

Adjusted for background information (age and gender) and related non-nutritive sucking habits (thumb sucking and pacifier use).

**The mean difference is significant at the 0.05 level.

^b^Adjustment for multiple comparisons: Bonferroni.

*P < 0.05.

Dependent variable: Intermolar width mm.

The duration of breastfeeding had no association on the development of a posterior crossbite in primary dentition (logistic regression: *P* = 0.358).

## Discussion

The present study reports the association of breastfeeding on three-dimensional dental-arch relationships in primary dentition. Children’s dental arch relationship was examined in three dimensions, and it was found that pure breastfeeding for more than 6 months benefits dental development in the anterior sagittal and transverse dimensions. These results agree with a study in Puerto Rico showed that children with a history of breastfeeding were associated with the development of a normal occlusion [18]. However, the Puerto Rico study did not explain in detail how breastfeeding benefits the development of primary dentition. Therefore, the findings in this study can further elaborate the findings from the Puerto Rico study.

In the sagittal dimension, we found that children who had experienced pure breastfeeding for more than 6 months had a significant lower chance of developing a class II incisal relationship and an increased overjet (greater than 3.5 mm) than those who had never been breastfed, although the duration of breastfeeding had no association on primary canine relationship, primary molar relationship, or anterior crossbite. Hence, the duration of pure breastfeeding mainly affects the primary incisor relationship but not the primary canine and primary molar relationships in primary dentition in the sagittal dimension. The results of this study revealed the association of duration of breastfeeding on the chance of developing Class II incisal relationship. Asian people present more convex profile than Caucasians and the prevalence of Class II in Chinese is 20.05% [31]. Therefore, it is important to promote breastfeeding as it is associated with reduction in Class II incisal pattern especially when we take into account the craniofacial growth pattern of the Asian population. The result of no association of breastfeeding with primary canine relationship agrees with the findings from US studies [1,15]. The benefits of breastfeeding are perhaps due to the different sucking motion involved in breastfeeding compared with bottle-feeding. Sucking a bottle requires the tongue to exert a piston-like action that compresses the artificial teats against the hard palate [1,14,32,33]. This motion causes a tongue thrusting action that is believed to cause an increased overjet in primary dentition. In contrast, both the nipple and areola are put into the mouth during breastfeeding and the tongue compresses the soft breast nipple in a peristaltic action to draw the milk. The movements of the lips and tongue result in a more squeezing rather than sucking action [14,32,33]. The benefits of breastfeeding may also be due to the fact that the breast is soft and can be easily adapted to the shape of the infant’s mouth, whereas the teats of bottles and pacifiers are harder and are much less yielding than the breast, forcing the infant’s mouth to change around them [32,33].

In the vertical dimension, the results of this study match those of previous studies in that there was no association between the duration of pure breastfeeding with anterior openbite or overbite in primary dentition [1,14,15]. Although a Brazilian study showed that children who were exclusively breastfed for more than 3 months had a higher chance of developing a favorable overbite, the study did not look into the association of non-nutritive sucking habits when analyzing the association of breastfeeding on the development of dental arch relationships [34]. Another reasons for the different results in different studies could be due to different standard in accessing overbite in deciduous dentition or due to different ethic group showing different characteristics of overbite in primary dentitions. Different sampling method could also contribute to the different results.

In the transverse dimension, this study found no association between the duration of pure breastfeeding and posterior crossbite, a finding that matches the results of previous studies from Italy and the US [1,15]. Furthermore, this study found that children who were purely breastfed for more than 6 months had greater intercanine and intermolar widths than those who were purely breastfed for less than or equal to 6 months. Hence, the duration of pure breastfeeding is directly proportional to the transverse dimension in primary dentition. These findings are likely to be due to the reduction in the posterior-acting forces of the buccinators during breastfeeding compared with bottle-feeding, pacifier use, and digit sucking habits [18]. Although there is a tendency that pure breastfeeding for more than 6 months may affect transverse dimension, the magnitude is limited. Moreover, the long-term maintenance in the transverse dimension cannot be concluded in this study and this warrants further investigations in future studies.

It is well documented that non-nutritive sucking habits such as pacifier use, digit sucking, and bottle-feeding are associated with malocclusions, including an increased overjet and a constricted upper arch [9-17]. To rule out the effects of non-nutritive sucking habits, this study took into account the possible association of the non-nutritive sucking habits, including pacifier use and thumb sucking on the development of the primary dental arches, and thus examined the genuine association of breastfeeding on the development of the primary dental arches.

Based on the recognition of nutritional and general health benefits of breastfeeding [1-3], the WHO recommends exclusive breastfeeding for up to 6 months of age [35]. In this study, the results suggested that exclusive breastfeeding with the duration recommended by the WHO also benefits primary dental development. Appropriate development of the masticatory muscles is stimulated by the constant sucking of the breast, as this action places great demand on the perioral musculature, creating suitable muscle tone that promotes correct oral functioning [36-38]. Even though it is still controversial, some studies show that a lack of breastfeeding or a short duration of breastfeeding favors the development of deleterious oral parafunctional habits because the child undertakes fewer oral exercises, which leads to the underdevelopment of the muscles and the improper positioning of the lip and tongue [36-44]. Taken into account the positive association of breastfeeding on dental development, the possible association of duration of breastfeeding on the development of parafunctional habits and the association of parafunctional habits on development of malocclusion [9-17,36-44], it could be assumed that children with non-nutritive sucking habits who are not breastfed for the first 6 months of life may tend to develop even more severe malocclusions, such as an overjet or posterior crossbite.

There are some limitations when evaluating the findings of this research. The samples were collected using cluster sampling, and there was 16% sample loss. There are 869 local kindergartens in Hong Kong and the distributions of kindergartens in the three territories, Hong Kong Island, Kowloon and New Territories are 19.5%, 30.4% and 50.1% respectively [45,46]. The territorial distributions of the 17 selected kindergartens are 2 in Hong Kong Island, 9 in Kowloon and 6 in New Territories. The distributions of the 10 participated kindergartens are 2 in Hong Kong Island, 7 in Kowloon and 1 in New Territories. Although the data in this study covered all three territories in Hong Kong, there may still exist some bias to certain group of population. Information on the duration of pure breastfeeding was obtained using retrospective questionnaires by the parents based on their memory, and thus the results are prone to recall bias and the habit data could not be validated. However, there is study that shows that maternal recall of breastfeeding even after twenty years after delivery was accurate [47]. According to the breastfeeding duration groups in this study, individuals exclusively breastfed for 6 months were included along with those individuals who breastfed exclusively for 1, 2 or 3 months. The results could have been different if the grouping had been distinct. Another limitation of this study is that this is a cross sectional study; hence, the cause and effect relationship of breastfeeding and three-dimensional dental arch relationships in primary dentition cannot be determined. So we can only assess the association. In the future, it is recommended that subjects with extensive carious lesion and loss of primary teeth should be excluded in the study as they may affect the occlusal development of primary dentition. Future examinations of the occlusal relationship of the children should be carried out with the subject sitting down rather than lying down as this may affect the position of the mandible relative to the maxilla. The occlusal measurements could be affected by different craniofacial pattern of the individual. Therefore, the individuals with distinct patterns such as the subjects with cleft, facial deformities or asymmetries were excluded from the study. However, children with mild class I, class II or class III skeletal patterns were not excluded, as it was not be possible to distinguish the children’s skeletal problem without a lateral cephalometric radiograph.

Most previous studies have merely assessed the unadjusted associations among the variables, and the majority were conducted in South America, North America or Europe. Also, only a few studies have systematically assessed the association of breastfeeding on the development of dental occlusions while taking into account confounding biases. This study thus contributes a valuable piece to the puzzle of how breastfeeding habits are associated with the development of primary dentition in Asians.

Current knowledge on the developmental occlusal relationship of the primary and permanent dentition is only limited to the primary and permanent molar relationship [21]. Therefore, further studies are required to investigate whether the changes in dental arch dimensions, for instance the incisor and transverse arch width, persist into mixed and permanent dentition. It is also needed to investigate the association between breastfeeding, oral habits, and malocclusions to generate a better picture of the development of dental occlusion.

The WHO recommends breastfeeding exclusively for 6 months with continued breastfeeding along with appropriate complementary food up to two years of age or beyond [35]. As the findings from this study show that pure breastfeeding for more than 6 months is associated with an increased arch width and a reduced overjet, it is valuable for the education and promotion of maternal breastfeeding. As this is the first study in Hong Kong to look into the association of breastfeeding on primary dental arch relationships, the results will be valuable for education and the promotion of maternal breastfeeding.

## Conclusion

Breastfeeding has positive association with dental arch development in primary dentition in both the anterior sagittal and transverse dimensions.

## Additional file

Additional file 1: **Oral Health Related Habits Questionnaire.** Questionnaires completed by the parents to collect information on the children’s duration of full-time breastfeeding, history of non-nutritive sucking habits (thumb sucking and pacifier use), as well as the family social economic status including their income and family background.
