# Supplementary material for: Association of breastfeeding and three-dimensional dental arch relationships in primary dentition
Source: BMC Oral Health. 2015 Mar 10;15:30. doi: 10.1186/s12903-015-0010-1 (PMC4358868; doi:10.1186/s12903-015-0010-1)
Supplement: Additional file 1: — Oral Health Related Habits Questionnaire. Questionnaires completed by the parents to collect information on the children’s duration of full-time breastfeeding, history of non-nutritive sucking habits (thumb sucking and pacifier use), as well as the family social economic status including their income and family background. [file 12903_2015_10_MOESM1_ESM.pdf]

Case Number:

Oral Health Related Habits Questionnaire:

Name of Child: _______________ Gender: ☐Male ☐Female
School's name: _______________ Parent's contact number: ________

1. Background information:
1.1 Child's birthday: ________Year _____Month _____Day

1.2 Child's place of birth: ☐Hong Kong ☐Mainland, China
☐ Others, Please specify: ___________

1.3Number of years living in Hong Kong: ________ Year(s)

1.4Does your child have the following medical conditions?
(Can choose more than one)
☐Congenital heart disease/ Rheumatic heart disease/ Other heart disease
☐Thalassemia/Hemophilia/ Other blood disease
☐Asthma/ Long term respiratory disease
☐Epilepsy
☐Long term use of medications, please specify:______________________________
☐Past surgical history, please specify: ______________________________________
☐Other important medical history, please specify : ___________________________

2. Para functional and feeding habits:
2.1 Has your child ever gotten a habit of thumb/digit sucking **ever since birth till now**?
☐Very often (Nearly every day) ☐Often (A few times a week)
☐Sometimes (A few times a month) ☐Not often ☐Never ☐Do not know

If with this habit, which finger does your child suck on?
☐Thumb ☐Second finger ☐Others(Please specify:____________)
(If answered "Never" or "Do not know" in question 2.1, please skip to question 2.4)

2.2 When did your child's thumb/digit sucking habit **start?**
☐0 to 6 months ☐6 months to 1 years old ☐1 to 2 years old
☐2 to 3 years old ☐3 to 4 years old ☐4 to 5 years old
☐5 to 6 years old ☐Do not know

2.3 When did your child's thumb/digit sucking habit **stop?**
☐0 to 6 months ☐6 months to 1 years old ☐1 to 2 years old
☐2 to 3 years old ☐3 to 4 years old ☐4 to 5 years old
☐5 to 6 years old ☐Still have this habit ☐Do not know

2.4 Has your child ever gotten a habit of using a pacifier **ever since birth till now**?
☐Very often (Nearly every day) ☐Often (A few times a week)
☐Sometimes (A few times a month) ☐Not often ☐Never ☐Do not know
(If answered "Never" or "Do not know" in question 2.4, please skip to question 2.8)

2.5 **On average**, how long does your child use the pacifier?
(Please specify: ________ hours a day)

2.6 When did your child's use of pacifier habit **start?**
☐0 to 6 months ☐6 months to 1 years old ☐1 to 2 years old
☐2 to 3 years old ☐3 to 4 years old ☐4 to 5 years old
☐5 to 6 years old ☐Do not know

2.7 When did your child's use of pacifier habit **stop?**
☐0 to 6 months ☐6 months to 1 years old ☐1 to 2 years old
☐2 to 3 years old ☐3 to 4 years old ☐4 to 5 years old
☐5 to 6 years old ☐Still have this habit ☐Do not know

2.8 Was your child **breastfed**?
☐Yes, entirely breastfed ☐Yes, partly breastfed and partly with powdered milk ☐No

2.9 How long was your child **entirely breastfed** for?
☐Less than 1 month ☐1 to 3 months ☐3 to 6 months ☐6 to 9 months
☐9 to 12 months ☐More than 12 months
☐Others: (Please specify:_________________________________)

3 Family background:
3.1 Your child is under a:
☐Single parent household ☐Two parent household
☐Others(Please specify:_________________________________)

3.2 Monthly **total** family income:
☐HK $ 10,000 or below ☐HK $ 10,001 -15,000
☐HK $ 15,001 -20,000 ☐HK $ 20,001 -25,000
☐HK $ 25,001 -30,000 ☐HK $ 30,001 -35,000
☐HK $ 35,001 or above

3.3 Parent's education level:
Father
☐Elementary school or below
☐Secondary school
☐Tertiary education/Collage or above

Mother
☐Elementary school or below
☐Secondary school
☐Tertiary education/Collage or above

6.4 Who **usually** take care of your child? (Please choose one)
☐Parents ☐Grandparents ☐Domestic helper
☐Others (E.g. Relatives or friends) Please specify: _______________

End of the questionnaire
Thank you for your participation!
